# Focused electron beam-induced deposition from Ru(CO)₄(fumaronitrile): incomplete carbonyl loss and nitrogen retention

**DOI:** 10.1140/epjd/s10053-026-01225-9

**Published:** 2026-08-05

**Authors:** Chinmai Sai Jureddy, Atul Chaudhary, Rashmi Singh, Lisa McElwee-White, Ivo Utke

**Affiliations:** 1 Empa - Swiss Federal Laboratories for Materials Science and Technology, 3603 Thun, Switzerland; 2 Department of Chemistry, University of Florida, Gainesville, FL 32611-7200 USA

## Abstract

The additive manufacturing of metal nanostructures using direct-write focused electron beam-induced deposition (FEBID) requires exploration and development of new metal-coordinated precursor complexes. In this study, we introduce (*η*^2^-fumaronitrile)(tetracarbonyl)ruthenium(0), a novel FEBID precursor designed using a mechanism-based approach in which the ligand set consists of neutral two-electron donor ligands weakly bound to the metal centre atom. Nanostructures were fabricated using this precursor via FEBID, and their elemental composition was determined through energy-dispersive X-ray spectroscopy. The resulting deposits consistently exhibited a metal content in the range of 8–12 at.%, highlighting the need for a deeper understanding of the role of competing mechanisms preventing detached neutral ligands from desorption during the FEBID process.

**Graphical abstract:**

**Figure:**
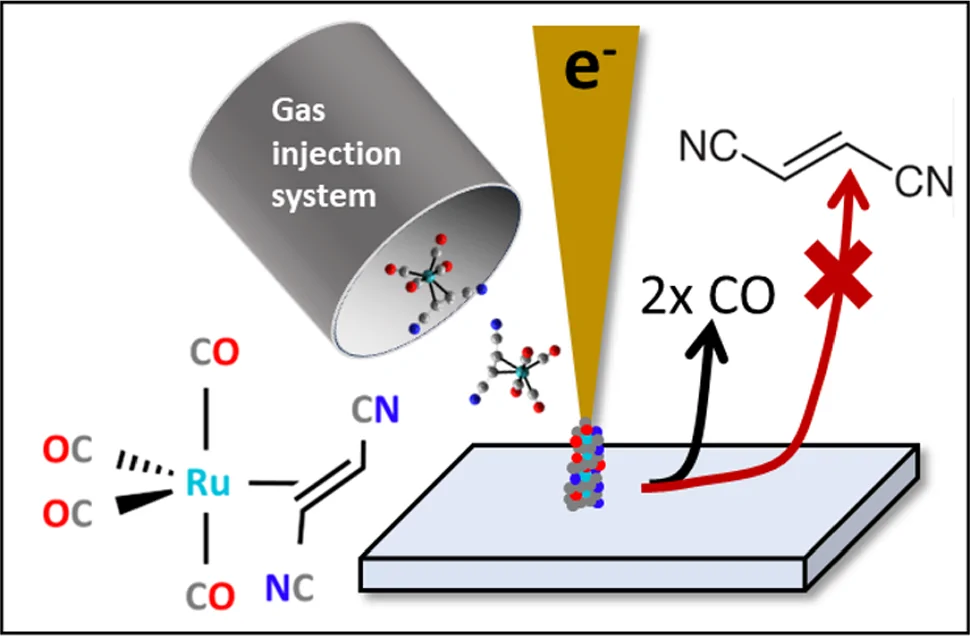
Fate of (*η*^2^-fumaronitrile)(tetracarbonyl)ruthenium in the focused electron-beam induced deposits

**Supplementary Information:**

The online version contains supplementary material available at https://doi.org/10.1140/epjd/s10053-026-01225-9.

## Introduction

Focused electron beam-induced deposition (FEBID) is an important additive nanofabrication technique due to its capability to directly write complex nanostructures [1–3]. This technique uses a bottom-up approach as compared to conventional lithography techniques. The process involves the localized dissociation of physisorbed precursor molecules on a substrate induced by an electron beam [4], resulting in material deposition. By tuning the electron beam scanning parameters, both two-dimensional and three-dimensional nanoprinted structures can be fabricated. Of particular interest is the fabrication of metallic nanostructures because of their relevance to advances in nanoscience and nanotechnology [5–10].

A wide range of metal–organic molecules have been explored as FEBID precursors and are commonly classified according to their ligand types. To date, the investigated ligand classes include carbonyls, halides, phosphines, amines, alkyls, allyls, *β*-diketonates, *β*-ketoesterates, carboxylates, and nitrosyls [1, 11, 12]. Most of these precursors were originally designed for chemical vapour deposition (CVD), often with chemical co-reactants, and are therefore not optimized for electron-driven fragmentation processes. A common example is the case of widely used (*ƞ*^5^-methylcyclopentadienyl)(trimethyl)platinum(IV), (*ƞ*^5^-MeCp)(Me)_3_Pt, where nearly 99 at.% Pt in the film [13] is observed in the CVD process while a maximum reported Pt content with the same precursor in FEBID is 28 at.% [14]. Therefore, a mechanistic design approach [15–17] for FEBID-specific precursors, based on the insights gained from electron irradiation studies of metal–organic molecules in the gas phase and in the condensed phase was proposed and as a result, new precursors are being developed. The precursors designed for FEBID include, Ru(*ƞ*^3^-C_3_H_5_)Br(CO)_3_ [18], CF_3_AuCNR [19] Ru(CO)_4_X_2_ (X = I, Br) [20], cis-Pt(Cl)_2_(NH_3_)_2_ [21], [Au(CH_3_)_2_Cl]_2_ [19], and carbonyl-based heterometallic precursors like (*η*^5^-C_5_H_5_)Fe(CO)_2_Mn(CO)_5_ [22], HFeCo_3_(CO)_12_, and H_2_FeRu_3_(CO)_13_ [23]. Until now, the deposition of high-purity metallic nanostructures (> 70 at.%) has been achieved for only a limited number of metals, partly due to an incomplete understanding of the underlying FEBID mechanisms, which differ from those operative under electron irradiation in gas phase and condensed phase conditions.

The processes occurring during FEBID involve not only electron-driven fragmentation but also simultaneous thermal processes, involving adsorption, desorption, and surface diffusion of molecules on the substrate surface. An overview of strategy towards increasing the metal content in FEBID deposits involving thermal and non-thermal mechanisms was described in Utke et al. [12]. In brief, achieving high metal purity requires not only the complete detachment of ligands from the metal centre, but it is also imperative that their desorption from the surface occurs before undergoing further electron-induced ligand fragmentation or polymerization leading to their residence on the surface. For example, carbonyl ligands are neutral and result in high metal contents when their bond energy to the metal centre is low (efficient detachment by electrons) [11, 24]. Precursors containing charged large ligands are more likely to simultaneously undergo intra-ligand fragmentation. This typically results in non-volatile and thus co-deposited organic ligand fragments [11, 12]. Exceptions comprise copper and palladium *β*-ketoesterates [25, 26] and silver carboxylate ligands [27–29]. The intra-ligand fragmentation seems to produce volatile moieties [30] that can readily desorb, which results also in deposited material metal contents > 70 at.%. FEBID studies with M(tert-butylacetoacetate)_2_ (M = Cu, Pd) have shown that fragments containing up to four carbon atoms, such as isobutylene, can desorb efficiently [25, 26]. In contrast, another study on FEBID using Ru(*ƞ*^3^-C_3_H_5_)Br(CO)_3_ exhibits negligible desorption of the C_3_H_5_ allyl ligand [18], owing to the anionic nature of allyl.

Based on these observations, ligands particularly promising for the design of FEBID precursors should have the following properties: they have facile dissociation of the metal–ligand bond, and they detach as neutral fragments from the metal centre, so they can desorb quickly. Ligands exhibiting these characteristics that have been explored for FEBID applications include CO, PX_3_ (X = F, H), NR_3_ (R = H, Me), benzene (C_6_H_6_), 3-dimethyl-1-butene (DMB), 2-methyl-1-hexen-3-yne (MHY) ([11, 12], and references therein), methyl acrylate (MA) [31], acrolein [32], and isonitriles of the general form CN-R [33]. Examples of precursors incorporating these neutral ligands are Cr(C_6_H_6_)_2_, Fe(CO)_4_MA, Ni(PF_3_)_4_, Pt(PF_3_)_4_ and the homoleptic carbonyl complexes. The formation of detached neutral ligands and ligand fragments upon electron impact is characteristic of neutral dissociation pathways. In mechanisms leading to charged fragmentation—specifically dissociative ionization (DI) and dissociative electron attachment (DEA)—the generation of neutral ligand fragments is likewise expected from the Stevenson–Audier–Harrison (SAH) rule [34–38] which implies that for a reaction *AB* + *e*^*−*^* → P*, *P* = *A* + *B*^+^*,* would be dominant over *P* = *A*^+^  + *B*, if fragment *B* has lower ionization potential than *A*. Gas phase experiments generally confirm this behaviour for metalorganics, producing charged metal-containing fragments alongside neutral ligand fragments [19, 20, 39–43] with few exceptions [44, 45]. Charged metal-containing fragments remain strongly bound to the surface due to high binding energies and long residence times and neutral ligands or ligand fragments can desorb more efficiently [46]. Hence, neutral ligands that can be efficiently removed by electron impact from the metal centre of the precursor, like the ligands mentioned above, and subsequently could easily desorb remain attractive candidates for FEBID precursor development.

**Fig. 1:**
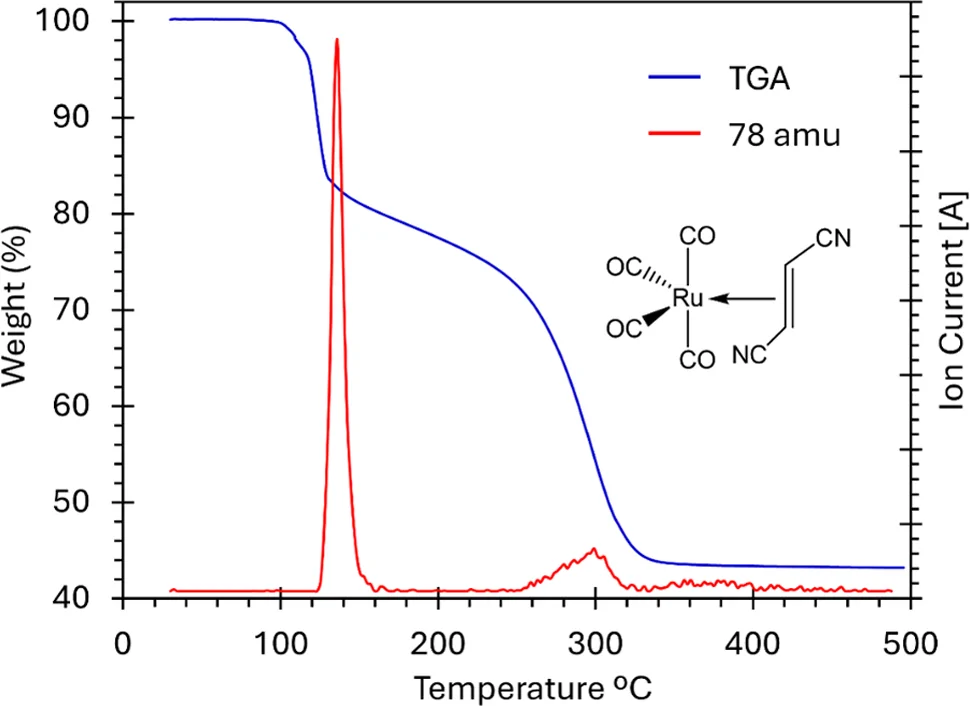
TGA–MS of Ru(CO)_4_FN showing the labile fumaronitrile ligand bond to the metal. The blue trace indicates the mass as a function of temperature. The red trace is the normalized intensity of the MS signal at *m/z* 78 corresponding to singly charged fumarontrile (C_4_H_2_N_2_)^+^ obtained by post-ionization of the neutral fumaronitrile ligand in the mass spectrometer

In this study, we investigate the behaviour of the fumaronitrile (FN, FN = (C_4_H_2_N_2_)) ligand in a newly designed heteroleptic ruthenium precursor, (*η*^2^-fumaronitrile)(tetracarbonyl)ruthenium, Ru(CO)_4_FN, see Fig. 1. The molecular structure of this precursor is shown in Fig. 1. FN interacts with the Ru centre via a bonding interaction between its *π*-bond and empty Ru orbitals, accompanied by *d*–π* backbonding, resulting in *η*^2^ coordination. As both FN and CO are neutral ligands, the ruthenium centre is formally in the zero-oxidation state [Ru(0)]. In addition, the fumaronitrile ligand–metal bond is particularly labile, as evidenced by combined thermogravimetric analysis and mass spectrometry (TGA–MS) of Ru(CO)_4_FN as shown in Fig. 1. In the TGA–MS, there is a strong MS peak during the first thermal mass loss at 140 °C at *m/z* 78, corresponding to fumaronitrile. Some concomitant CO loss (*m/z* 28) during the first mass loss is also detected by TGA–MS under Ar but could not be quantified due to leaks of N_2_ (also *m/z* 28) during measurement. Loss of the fumaronitrile *η*^2^-alkene ligand from the metal competes well with CO loss, which is atypical of complexes with both *π*-facial ligands and carbonyls. Moreover, FN has zero net dipole moment which could allow it to have higher desorption rate. We report the tailored deposition conditions optimized for this precursor and discuss the resulting deposit composition for FEBID.

## Methods

### Precursor synthesis

All of the reactions were carried out under an inert atmosphere of dinitrogen using Schlenk line techniques [47]. Glassware was flame-dried or oven-dried before use. Solvents were purified using an MBraun MB-SP solvent purification system and stored over 3 Å molecular sieves before use. Reagents were purchased from either MilliporeSigma or Oakwood Chemicals and used without further purification. Ethylene (CP grade) was purchased from Airgas. Deuterated solvents for NMR were purchased from Cambridge Isotopes Lab and used without further purification. Silica gel used for chromatography was used as received. ^1^H NMR nuclear magnetic resonance spectra (NMR) were obtained on a Bruker 400 MHz spectrometer. All chemical shifts are reported in *δ* (ppm) and referenced to residual proton peaks in the solvent. IR spectra were obtained on a PerkinElmer Spectrum ONE FT-IR spectrometer using a solution cell equipped with NaCl windows and a path length of 1.0 mm.

The Ru(CO)_4_FN compound was synthesized using a modified literature procedure [48]. A solution of Ru_3_(CO)_12_ (0.35 g, 0.56 mmol) in anhydrous hexanes (200 mL) was added to a 500 mL three-necked round bottom flask. The solution was irradiated with blue LED light (450 nm) under an ethylene atmosphere with slow bubbling for 2 h at room temperature. The reaction turned clear after irradiation for 2 h and the gas inlet was switched from ethylene gas to dinitrogen to reduce the concentration of ethylene. Fumaronitrile (0.14 g, 1.79 mmol) was added to this solution and after 30 min white precipitate was formed. The crude product was obtained by the removal of the solvent using a rotary evaporator. The crude product was directly purified by silica gel chromatography (DCM:n-hexanes = 90:10) and obtained as an off-white coloured product (90.00 mg, 55%) and characterized by comparison to literature data [48]. IR (DCM, cm^−1^): 2044, 2075, 2143, 2219. ^1^H NMR (400 MHz, CDCl_3_) *δ* 2.73 (*s*, 2H).

### Thermogravimetric analysis/mass spectrometry

Spectra were obtained using a residual gas analyzer (RGA) mass spectrometer (Cirrus 3) connected to a TGA Discovery 5500. Samples of 1–5 mg were placed in an alumina crucible and heated from 20 to 500 °C at a rate of 10 °C min⁻^1^. A heated transfer line carried volatile gases evolved during thermal decomposition to the mass spectrometer. The transfer line was set within the range of 100–200 °C, and the analyses were performed under vacuum conditions of approximately 10^–6^–10^–5^ Torr. Mass spectrometry was operated at 70 eV, and a scan was performed from 1 to 300 amu. Measurements of the *m/z* 78 signal (fumaronitrile) were performed under inert N_2_ conditions. Measurements of the *m/z* 28 signal (CO) were performed under Ar but the background signal from N_2_ leaks in the instrument could not be eliminated.

### Deposition experiments and characterization

FEBID experiments were carried out using a Hitachi S3600 and a Philips XL30 scanning electron microscope (SEM). Native oxide covered Si(100) substrates were used for all depositions. The precursor was introduced into the chamber via a heatable, custom-built gas injection system (GIS) and was loaded under ambient conditions. Square deposits were fabricated using the built-in electron beam scanning systems of the SEMs. The impinging electron current was measured by a Faraday cup and the electron flux determined by the full width at half maximum size of the Gaussian electron beam. The molecule throughput was determined by mass loss measurements of the reservoir before and after the experiments. The conversion to molecule flux arriving at the substrate was performed by taking into account the geometrical arrangement of the GIS with the substrate using the GISsimulator software described in Friedli and Utke [49].

Elemental composition was determined by energy-dispersive X-ray spectroscopy (EDX) using a silicon drift detector (Oxford Instruments), with an electron beam energy of 6 keV. Deposit morphology was examined using high-resolution secondary electron imaging in a Hitachi S4800 field-emission SEM. Atomic force microscopy (AFM) measurements were performed with an NT-MDT NTEGRA Spectra system to determine deposit thickness. This thickness information was used to correct the compositional analysis by accounting for substrate signal contributions, where necessary using thin-film corrections software, SAMx Stratagem [25, 28]. To investigate the influence of FEBID parameters on the composition and morphology, depositions were performed under different substrate temperatures (25–80 °C), GIS temperatures (50–70 °C), electron beam currents (0.1–14 nA), and electron beam energies (10–25 keV).

## Results and discussion

### Deposition conditions and deposit morphologies

**Fig. 2:**
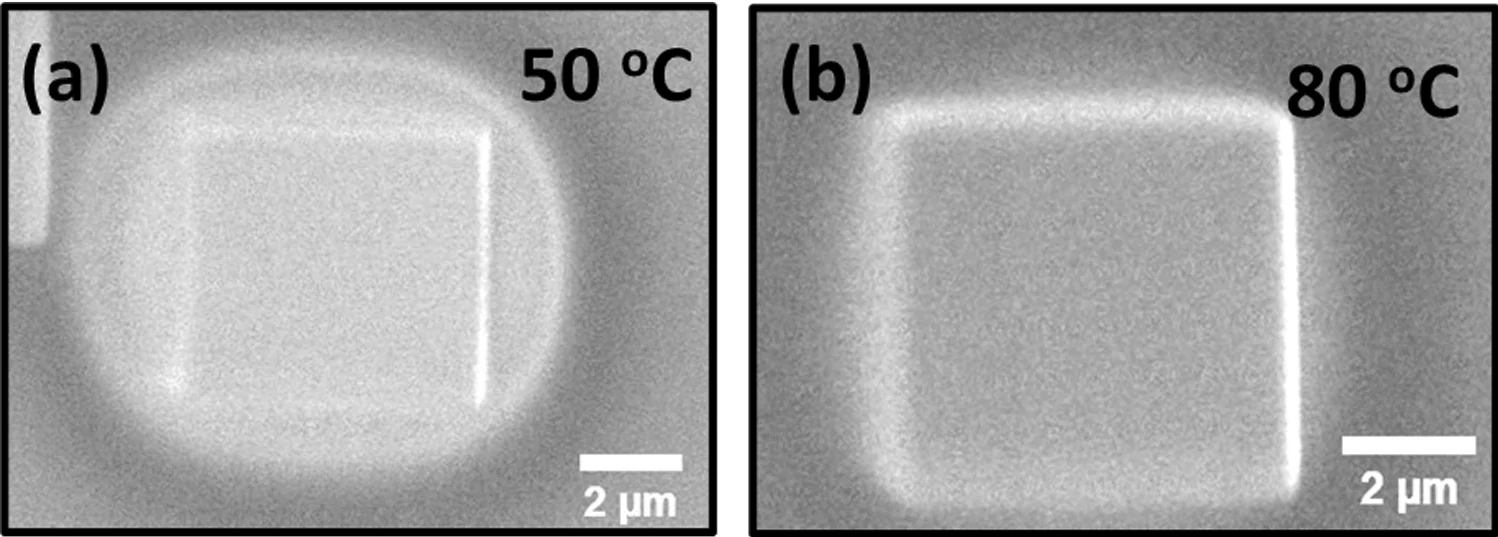
Top view secondary electron SEM images of two 5.071 µm × 5.071 µm FEBID squares deposited at the same beam current of 0.5 nA using a 20 keV electron beam with *T*_GIS_ = 70 °C and **a** *T*_sub_ = 50 °C, **b** *T*_sub_ = 80 °C. A smaller halo extent region at higher substrate temperature is observed due to increased thermal desorption of precursor molecules

Figure 2a presents a secondary electron (SE) image of a square FEBID deposit. The deposit, with lateral dimensions of 5.071 × 5.071 µm^2^, was fabricated by scanning a 20 keV electron beam using a dwell time of 1 µs and a pitch of 4.9 nm over a total deposition time of 20 min, corresponding to 1145 scan cycles at a beam current of 0.5 nA as measured on the substrate. The gas injection system and substrate temperatures were maintained at *T*_GIS_ = 70 °C and *T*_sub_ = 50 °C, respectively. A pronounced round halo region surrounding the main square deposit is observed (bright contrast), which arises from deposition induced by backscattered electrons (BSEs) and secondary electrons generated by BSEs. The presence of a halo region signifies that the precursor may have a relatively high cross-section for electron-induced fragmentation compared to those precursors which do not exhibit a halo deposit region. Increasing the substrate temperature to 80 °C (Fig. 2b) leads to a significant reduction of the halo deposit, which we primarily attribute to the enhanced desorption rate of the precursor at elevated substrate temperatures since all the other deposition parameters were the same.

**Fig. 3:**
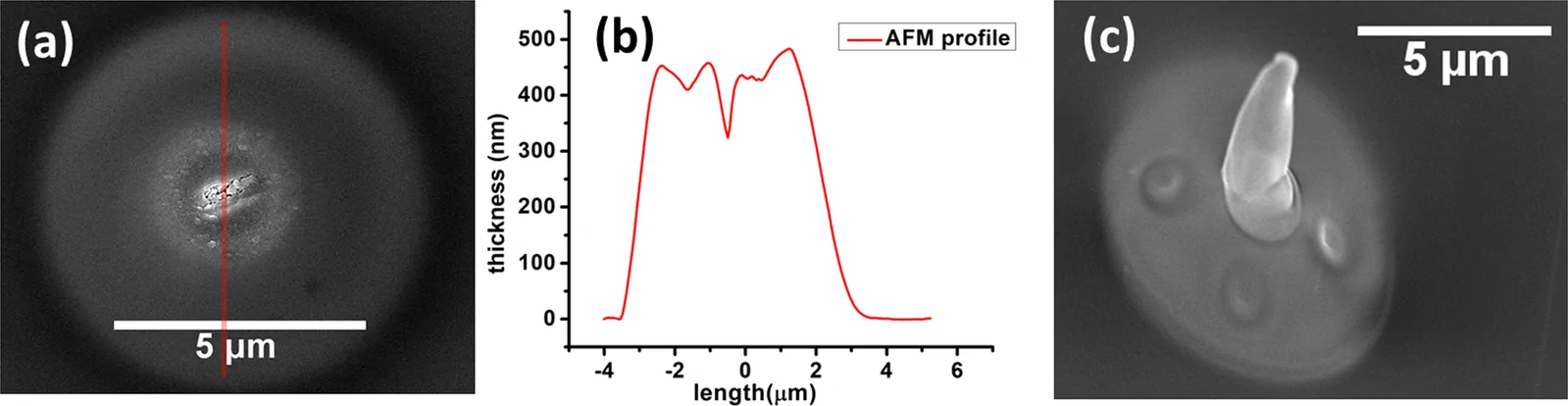
**a** Top view SEM image of FEBI spot deposit performed at *T*_GIS_ = 50 °C and substrate at room temperature for the deposition time of 90 min. **b** AFM profile across the centre of the deposit, shown as red line in **a**. **c** SEM tilt image of a FEB spot deposit at *T*_GIS_ = 70 °C and *T*_sub_ = 50 °C for the deposition time of 15 min. See text for the details of molecule and electron fluxes

The precursor is also capable of producing deposits on a room-temperature substrate with *T*_GIS_ = 50 °C. Figure 3a shows a spot deposit fabricated under these conditions with the electron beam current of 0.8 nA as measured on the Faraday cup at 20 keV energy. The molecule arrival rate was 3.6 × 10^14^ molecules/s which translates to a molecule arrival flux of 6.7 × 10^7^ molecules/µm^2^s at the substrate in the deposition areas; the electron flux was 2.5 × 10^8^ electrons/µm^2^s. A granular structure is observed at the centre of the deposit, appearing as a bright region in the SE image, indicating a significant variation in deposit thickness. AFM measurements taken along the red line in Fig. 3a confirm the presence of a central depression (see Fig. 3b). We also observed the grain size decreased from the centre. Figure 3c shows the similar spot deposit for deposition conditions of *T*_GIS_ = 70 °C and *T*_sub_ = 50 °C, and a beam current of 0.53 nA as measured with Faraday cup. In this case, the molecule arrival rate was 1.9 × 10^17^ molecules/s, i.e., the molecule arrival flux on the substrate was 3.5 × 10^10^ molecules/µm^2^s and the electron flux of 7.3 × 10^8^ electrons/µm^2^s. Comparing the vertical deposition rates, in the former case, it is approximately 0.08 nm/s where as in latter case, it is around 3.5 nm/s which corresponds to a significant enhancement by a factor of 44.

### Deposit composition

**Fig. 4:**
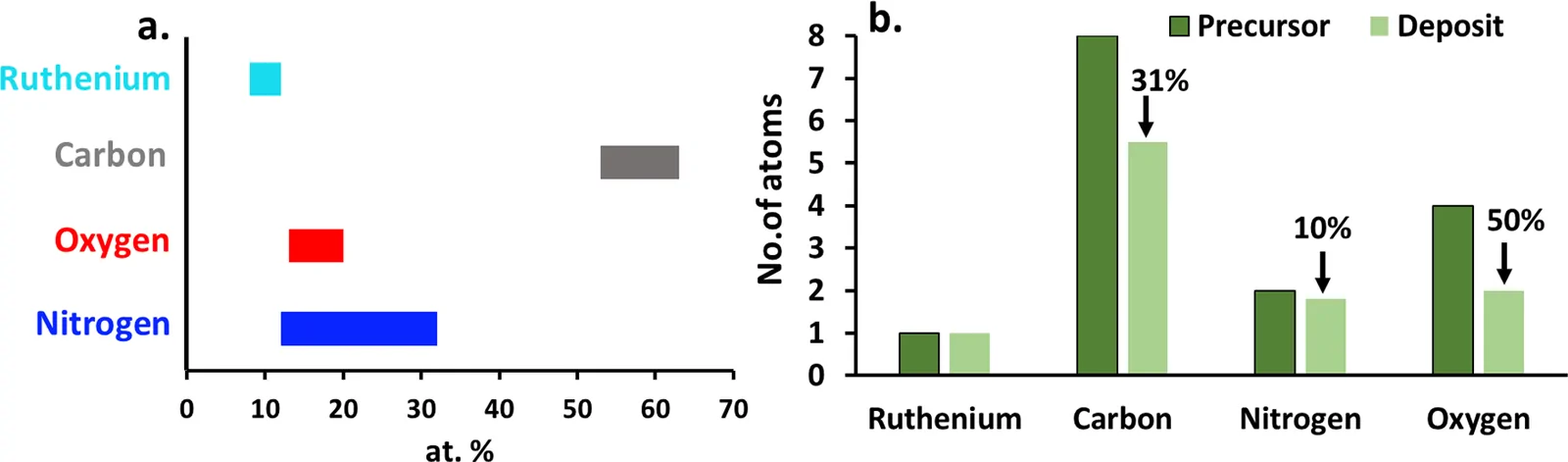
**a** EDX measured composition range of FEBID material deposited using various FEBID parameters, for details see text. **b** Comparison of atomic ratios of precursor versus the FEBID deposit in Fig. 2a. The composition is represented by the number of atoms of Ru, N, C, and O, normalized to one Ru atom. Element loss is indicated by arrows. Note the low loss of nitrogen atoms. Hydrogen is not included as it cannot be detected through EDX

The compositions were determined from EDX measurements neglecting the hydrogen content. The ruthenium content of the deposit as shown in Fig. 2a is 9.4 at.%, compared to 6.67 at.% in the pristine precursor. For comparison, the highest reported Ru content achieved via FEBID is 52 at.% using the precursor Ru(CO)_4_I_2_ [50]. Other Ru precursors include bis(*η*^5^-ethylcyclopentadienyl)ruthenium(II), Ru(EtCp)_2_, and Ru(*η*^3^-C₃H₅)(CO)₃Br, which yield Ru contents of 10 at.% and 23 at.%, respectively [18, 51]. Increasing the substrate temperature to *T*_sub_ = 80 °C does not result in an increase in Ru content, and the halo regions exhibit a composition similar to that of the main deposit (see Supporting Information Figure S1). Furthermore, deposits fabricated under different primary electron beam energies (10–25 keV), beam currents (0.1–14 nA), substrate temperatures (50–80 °C), and GIS temperatures (40–70 °C) consistently show Ru contents between 8 and 12 at.% as summarized in Fig. 4a (see Supporting Information for details).

The atomic ratio Ru:C:O:N of the FEB deposit of Fig. 2a is 1:5.7:2.1:1.8. Hydrogen atoms are not considered as they cannot be detected by EDX measurements. Figure 4b compares the atomic concentrations of C, N, and O in the deposit with those in the precursor. This elemental analysis indicates the loss of approximately two oxygen atoms and about two carbon atoms per precursor molecule, and ≈ 0.4 atoms of nitrogen. Overall, the fraction of ligand atoms removed during deposition is relatively low, amounting to approximately 33% removal of the pristine ligand atoms. We performed deposits using electron beam energies between 10 and 25 keV, across a range of beam currents and substrate temperatures, but no significant compositional differences are seen, aside from morphological variations (see Supporting Information section S2). Increasing the molecular flux led to an increase in the deposition rate, again with no significant compositional difference.

From the measured deposit composition range, the observed loss of carbon and oxygen atoms can be attributed to the removal of approximately 2–2.5 carbonyl ligands per each precursor molecule (containing four CO ligands initially). The as-deposited structures obtained from Ru(*η*^3^-C_3_H_5_)(CO)_3_Br, deposited under conditions comparable to those used in the present study (20 keV beam energy, 0.65 nA beam current measured on Si, and room-temperature substrate), exhibit complete removal of all three carbonyl ligands [18]. For the Ru(II) complex Ru(CO)_4_I_2_, FEBID performed with a 5 keV electron beam under ultra-high vacuum conditions resulted in an average loss of approximately 3.5 CO ligands per precursor molecule [50]. One possible contributing factor is the electron density at the ruthenium centre, which controls the amount of *d*–π* backbonding to the *π*-acidic CO ligands, and thus the Ru–CO bond strength. Because metal carbonyls have found extensive use as reporter ligands for electron density at metal centres [52], information on the correlation of their IR stretching frequencies to bond strengths, spectroscopic observations and reactivity is readily available [53–55]. Lower CO stretching frequencies are indicative of higher metal-CO bond strengths. Comparison of the lowest vibrational frequencies for CO stretching in Ru(CO)_4_FN (2044 cm^−1^) and Ru(CO)_4_I_2_ (2066 cm^−1^) is consistent with stronger Ru–CO bonding in Ru(CO)_4_FN, and hence less CO loss during FEB irradiation. In addition, reactions of liberated CO with radical or ionic species generated on the surface during electron irradiation may contribute to the retention of carbon and oxygen within the deposit. At present, the relative importance of these effects cannot be established.

Focusing on the nitrogen content in the FEB deposits, we observe almost no decrease of the number of nitrogen atoms as compared to pristine precursor. When considered alongside the carbon content, this suggests that the fumaronitrile (FN) ligands either undergo negligible detachment from the metal centre or possess a low desorption rate sufficient to allow for their further electron-induced decomposition into non-volatile species on the surface. Negligible fumaronitrile detachment from the metal atom is very unlikely in view of the TGA–MS analysis as shown in Fig. 1, showing a high propensity for the FN loss already at thermal energies and indicating a weak bond with the metal atom. However, although being a neutral ligand and thus eligible for easy desorption, fumaronitrile contains cyano groups with localized dipole moments (although having zero net dipole moment) which may result in longer residence times on the surface. Therefore, with respect to nitrogen retention, the co-dissociation and co-deposition of FN is a plausible explanation. This co-dissociation might involve the formation of HCN, as suggested by electron impact ionization studies of gas phase fumaronitrile [56]. The removal of nitrogen through HCN could be a plausible explanation for the observed loss of ≈ 0.4 N atoms in some of the FEB deposits. A similar co-deposition of nitrogen-containing ligands was observed for CF_3_AuCNMe and CF_3_AuCN^*t*^Bu, which reported no loss of nitrogen upon FEB deposition, indicating decomposition of the ligand rather than efficient desorption when performed under UHV conditions with 3 keV electron energy [33].

A structurally related neutral organic ligand, methyl acrylate (MA, MA: H_2_C=CH–COOCH_3_) investigated in Fe(CO)_4_MA, exhibited efficient ligand loss during electron beam-induced deposition at 100 eV due to the absence of electron-induced polymerization pathways [31]. Thermal desorption of MA after electron-induced detachment from Fe(CO)_4_MA was concluded to be a feasible pathway for the removal of carbon from the deposit. Furthermore, MA has vapour pressure of 90.5 mbar at 20 °C which is more than 100 times higher than that of FN, whose vapour pressure is 0.85 mbar at 25 °C. This comparison suggests that, although FN is a neutral ligand, its relatively low desorption rate may limit its suitability for FEBID precursor design. The important role of high desorption rates of detached ligands versus their dissociation rate was recently quantitatively modelled in Jurczyk et al. [57] for obtaining high metal content FEBID material. Ligand rate ratios of desorption versus dissociation << 1 will always result in low metal content unless orders of magnitude decrease in electron flux or residence time (exponential temperature dependence) can be achieved. These changes, however, may also come along with unacceptable low FEB deposition rates. The variation of FEBID parameters performed in our study still giving observable deposition rates did not result in significant composition changes.

Finally, we would like to point out that hydrocarbon-based ligands generally exhibit higher vapour pressures than oxygen- or nitrogen-containing organic ligands. They may thus represent more favourable ligand candidates for FEBID precursors. A direct comparison can be drawn from the behaviour of MHY and DMB ligands in Cu(hfac)L (L = MHY, DMB) complexes [58]. Although detailed dissociation pathways were not discussed, the FEBID deposit composition indicates a loss of approximately 8.6 carbon atoms from the pristine Cu(hfac)(DMB) molecule, which suggests the cleavage and subsequent desorption of the DMB ligand. On this basis, future studies employing zero valent precursors such as (*η*^4^-butadiene)Ru(CO)_3_ and (*η*^4^-isoprene)Ru(CO)_3_ [59] may provide further insight into the role and applicability of neutral hydrocarbon ligands in FEBID precursor design.

## Conclusion

Achieving nanostructures with high metal purity via FEBID requires a detailed understanding of the role played by different ligand types in precursor fragmentation and desorption. Neutral ligands, which generally exhibit weaker interactions with the metal centre than anionic ligands and possess higher desorption rates, are therefore of particular interest for FEBID applications. In this study, we investigated the heteroleptic precursor (*η*^2^-fumaronitrile)(tetracarbonyl)ruthenium, Ru(CO)_4_FN, composed entirely of neutral ligands, including a labile *η*^2^-FN. FEBID-fabricated deposits produced under a range of deposition conditions exhibit ruthenium contents between 9 and 12 at.%. Analysis of the deposit stoichiometry indicates the loss of approximately only two carbonyl ligands, with nitrogen largely retained in the deposits. The reason for the remaining two carbonyl ligands retention is currently unclear. We attribute the absence of nitrogen loss to the long surface residence time of the FN ligand, arising from its locally polarized cyano groups and its tendency to decompose rather than desorb, similar to behaviour observed for CF_3_AuCNR (R = methyl, tert-butyl) precursors. This further emphasizes the critical role of rapid desorption of detached ligands in FEBID to obtain metal rich deposits, though the ligands prove to be labile for cleavage from the metal centre. This work also demonstrates that ligands with no net dipole moment could still have long residence times depending on their functional groups, nitriles in this case. Based on these findings, neutral hydrocarbon ligands such as isoprene and butadiene, which possess higher desorption rates than cyano-containing organic ligands, may represent more promising candidates for future FEBID precursor design.

## Supplementary Information

Below is the link to the electronic supplementary material.

Supplementary file1 (PDF 264 kb)

## Data Availability

This manuscript has associated data in a data repository. [Author′s Comment: Data will be available upon request from the corresponding author.]
