# Supplementary material for: Focused electron beam-induced deposition from Ru(CO)₄(fumaronitrile): incomplete carbonyl loss and nitrogen retention
Source: Eur Phys J D At Mol Opt Phys. 2026 Aug 5;80(8):115. doi: 10.1140/epjd/s10053-026-01225-9 (PMC13437608; doi:10.1140/epjd/s10053-026-01225-9)
Supplement: Supplementary file 1 [file 10053_2026_1225_MOESM1_ESM.pdf]

Supporting information

S1: Composition in the halo region

The composition obtained at the centre and in the halo were compared for the deposit reported in figure 2a in the main text. In the halo region, the Si contribution was not eliminated for this deposit. Nevertheless, the composition after eliminating the Si content would be expected to be to lie in the range of the other deposits.

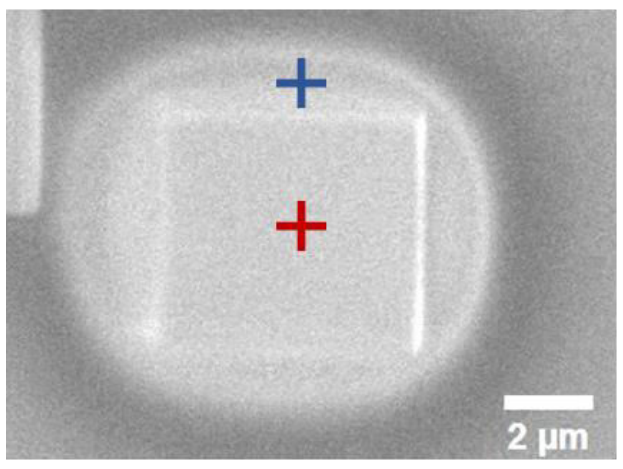

Element	Composition from EDX (at.%)	
	centre	halo
Ru	9	6
C	54	54
N	17	14
O	20	16
Si	0	10

Figure S1: The composition obtained from EDX measurements performed at the centre (red) and in the halo region (blue) are tabulated.

S2: Compositions of all deposits fabricated

Here, we report the composition of all the deposits fabricated under different conditions.

A) Spot depositions using 20 keV electron beam with 0.8 nA beam current. The substrate is at room temperature and T_{GIS} = 50 °C

	Elemental composition			
**Deposition time**	**Ru** Atoms/at %	**C** Atoms/at %	**N** Atoms/at %	**O** Atoms/at %
30 min	1/10.4	5.6/58.3	1.5/15.6	1.5/15.6
60 min	1/10.6	5.7/60.6	1.3/13.8	1.4/14.9
90 min	1/10.9	5.3/57.6	1.4/15.2	1.5/16.3
Precursor	1/6.67	8/53.56	2/13.34	4/26.68

B) Spot depositions using 20 keV electron beam. The substrate is at room temperature and T_{GIS} = 50 °C. The deposition time is 90min.

**Current** (as measured on faraday cup) in nA	Elemental composition			
	**Ru** Atoms/at %	**C** Atoms/at %	**N** Atoms/at %	**O** Atoms/at %
0.8	1/10.9	5.3/57.6	1.4/15.2	1.5/16.3
0.4	1/10.4	5.6/58.3	1.5/15.6	1.5/15.6
0.2	1/10.0	6.0/60.0	1.4/14.0	1.6/16.0
0.1	1/8.2	7.4/60.7	1.7/13.9	2.1/17.2
precursor	1/6.67	8/53.56	2/13.34	4/26.68

C) Spot depositions at different electron beam energies with beam current of 0.2nA. The substrate is at room temperature and T_{GIS} = 50 °C. The deposition time is 60min.

	Elemental composition			
**Electron energy (keV)**	**Ru** Atoms/at %	**C** Atoms/at %	**N** Atoms/at %	**O** Atoms/at %
15	1/8.8	7.4/64.9	1.5/13.2	1.5/13.2
20	1/8.9	7.0/62.5	1.5/13.4	1.7/15.2
30	1/8.0	8.0/64.0	1.6/12.8	1.9/15.2
precursor	1/6.67	8/53.56	2/13.34	4/26.68

D) Depositions carried out at different substrate temperatures at 20keV electron beam energy.

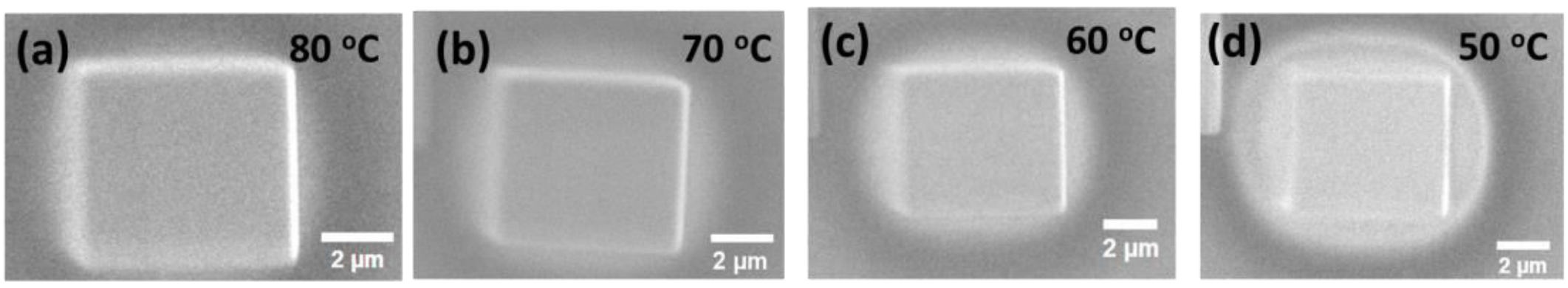

	At.% / atoms
C	53.0 / 5.3
N	15.8 / 1.6
O	20.7 / 2.1
Si	0.5 / 0.06
Ru	10 / 1

	At.% / atoms
C	53.9 / 5.4
N	15.1 / 1.5
O	20.6 / 2.1
Si	0.4 / 0.04
Ru	10 / 1

	At.% / atoms
C	52.7 / 5.7
N	17.6 / 1.9
O	20.0 / 2.2
Si	0.5 / 0.06
Ru	9.2 / 1

	At.% / atoms
C	53.5 / 5.7
N	17.1 / 1.8
O	20.1/ 2.1
Si	0.00 / 0
Ru	9.4 / 1

Figure S3: FEBI deposits carried out at T_{GIS} = 70°C and at different substrate temperatures (a) T_{sub} = 80°C, (b) T_{sub} = 70°C, (c) T_{sub} = 60°C, (d) T_{sub} = 50°C. Their corresponding compositions are tabulated below.

E) Depositions carried out at different electron beam current at 20 keV electron energy.

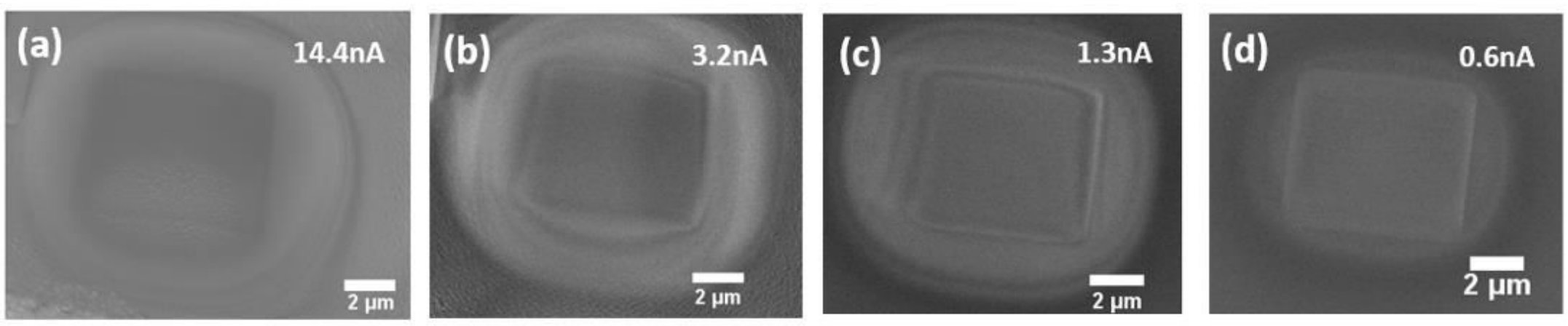

	At.% / atoms
C	54.3 / 4.6
N	19.3 / 1.6
O	14.7 / 1.3
Ru	11.7 / 1

	At.% / atoms
C	53.7 / 5.3
N	16.6 / 1.6
O	19.2 / 2.1
Ru	10.5 / 1

	At.% / atoms
C	53.9 / 5.3
N	17.9 / 1.6
O	18.7 / 2.1
Ru	9.5/ 1

	At.% / atoms
C	57.4 / 5.3
N	14.9 / 1.6
O	18.2 / 2.1
Ru	8.8/ 1

Figure S4: FEBI deposits carried out with T_{sub} = 50 °C and T_{GIS} = 70 °C at different electron beam currents (a) 14.4 nA, (b) 3.2 nA, (c) 1.3 nA, and (d) 0.6 nA. Their corresponding compositions are tabulated below.
